# Analysis of Demographic Characteristics of Users of a Free Tobacco Cessation Smartphone App: Observational Study

**DOI:** 10.2196/32499

**Published:** 2022-03-09

**Authors:** Nick Fradkin, Susan M Zbikowski, Trevor Christensen

**Affiliations:** 1 Office of Healthy and Safe Communities Division of Prevention and Community Health Washington State Department of Health Olympia, WA United States; 2 2Morrow, Inc Kirkland, WA United States

**Keywords:** mobile applications, mHealth, eHealth, smartphone app, tobacco, smoking cessation, public health, smoking, application

## Abstract

**Background:**

Tobacco use continues to be the leading preventable cause of death, disease, and disability in the United States. Since 2000, Washington state has offered free tobacco “quitline” services to help its residents stop using tobacco. In 2015, the state began offering free access to a tobacco cessation smartphone app to absorb excess quitline demand. Since most publicly funded tobacco cessation programs are designed to provide access to populations disproportionately impacted by tobacco use, it is important to consider who these public health interventions reach.

**Objective:**

The aim of this study is to understand who used a free cessation app and the extent to which users represented populations disproportionately impacted by tobacco use.

**Methods:**

This is an observational study of 1280 adult Washington state residents who registered for and activated the cessation app. Demographic data were collected as part of the sign-up process, examined using standard descriptive measures, and assessed against state-level surveillance data for representativeness.

**Results:**

Participants were primarily non-Hispanic White (978/1218, 80.3%), identified as female (780/1236, 63.1%), were between ages 25-54 years (903/1186, 76.1%), had at least some college education (836/1222, 68.4%), and reported a household income under US $50,000 (742/1055, 70.3%). Fewer respondents were from rural counties (359/1220, 29.4%); identified as lesbian, gay, bisexual, pansexual, queer, questioning, or asexual (LGBQA; 153/1222, 12.5%); were uninsured (147/1206, 12.2%); or were currently pregnant, planning pregnancy, or breastfeeding (42/624, 6.7%). However, relative to available state data for tobacco users, there was high representation of women, 35- to 54-year-olds, college graduates, and LGBQA individuals, as well as individuals with low household income, poor mental health, Medicaid insurance, and those residing in rural counties.

**Conclusions:**

A diverse population of tobacco users will use a free cessation app, including some demographic groups disproportionately impacted by tobacco use. With high reach and high efficacy, it is possible to address health disparities associated with tobacco use and dependence treatment among certain underserved and at-risk groups.

## Introduction

Smoking and secondhand smoke exposure in the United States lead to approximately 480,000 deaths each year [1], and tobacco use continues to be the leading preventable cause of death, disease, and disability [2]. It is widely accepted that there is no safe level of cigarette smoking [3] and that smoking cessation has benefits at any age [2,4].

Among US adults in 2019, cigarette smoking (14%) was the most common form of tobacco use, followed by electronic cigarettes (e-cigarettes; 4.5%), cigars (3.6%), smokeless tobacco (2.4%), and pipes (1%) [5]. In Washington state (WA), nearly 1 in 5 (17.4%) [6], or 1 million, adults use one or more of these tobacco products [7]. In WA, as in the US, adults who use tobacco are disproportionately male; lesbian, gay, bisexual, pansexual, queer, questioning, or asexual (LGBQA); have low educational attainment; have low household income; have poor mental health; and live in rural areas [5,6].

Over the last several decades, the rate of smoking has declined largely due to tobacco control policies such as tobacco taxes, smoke-free workplaces and spaces, public awareness campaigns, and the availability of effective cessation treatments [8]. Cessation options have been available direct-to-consumer and through employers, health insurance plans, and publicly sponsored programs. One such state-sponsored program is tobacco “quitlines,” for which there is a large body of supporting evidence [9]. Quitlines, which typically offer free phone counseling, web-based resources, and cessation medications [10], represent a cost-effective, population-based approach for reducing tobacco-related disease and death [8], and are available in all states and, increasingly, internationally [11].

In 2000, the WA Department of Health (DOH) started one of the first state quitlines in the United States [12] and continues to offer free evidence-based cessation resources to residents. Following the loss of state quitline funding, the DOH began offering free access to a smartphone app in 2015 to absorb excess quitline demand and reach a broader audience with cessation services. In this paper, we describe the demographic characteristics of adults who activated a tobacco cessation app, and compare these demographics to those of the broader WA adult tobacco user population to understand the extent to which populations disproportionately impacted by tobacco use have used the app.

## Methods

### Background and Ethical Approval

The present study is a real-world observational study based on data from 1280 WA residents who registered for and activated the 2Morrow Health Smoking & Tobacco app (2Morrow Inc) between October 1, 2018, when the DOH last updated its sign-up process and questions, through December 31, 2020. Demographic data were collected as part of the sign-up process (described below), examined using standard descriptive measures, and compared to the overall WA tobacco user population. The Washington State Institutional Review Board determined that this study did not constitute human subject research and was deemed exempt from the associated ethical requirements (2021-044).

### App Description

**Figure 1:**
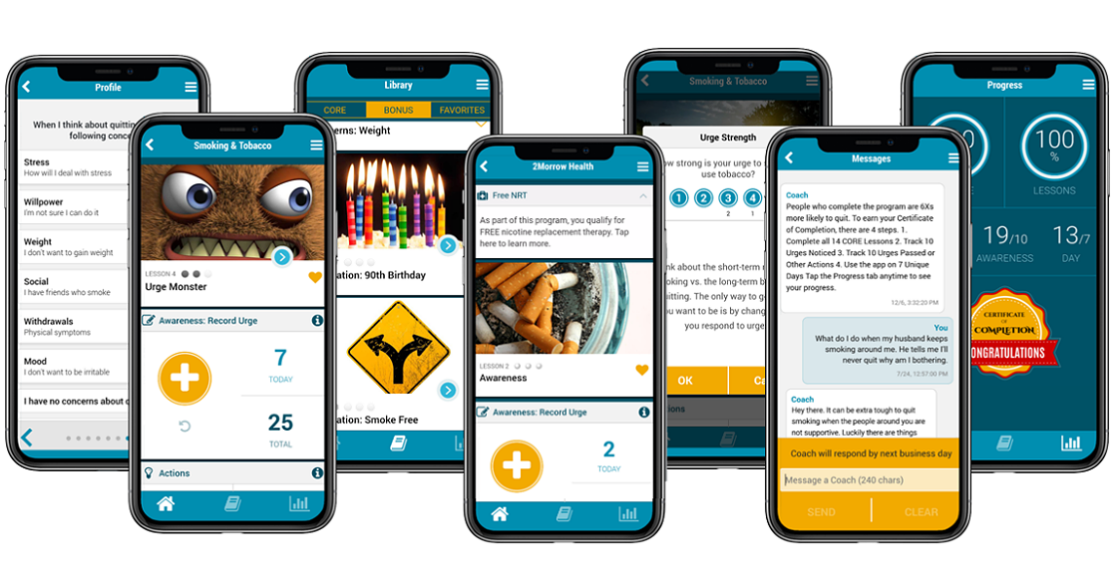
2Morrow Health Smoking & Tobacco app. NRT: nicotine replacement therapy.

The 2Morrow Health Smoking & Tobacco app (Figure 1) is a self-guided smartphone app designed to teach tobacco users how to manage unhelpful thoughts, urges, and cravings caused by nicotine addiction. The app was originally developed and tested by researchers at the Fred Hutchinson Cancer Research Center in Seattle, WA, and brought to market by 2Morrow Inc through an exclusive licensing arrangement. The app is evidence-informed, grounded in acceptance and commitment therapy principles, and scientifically tested [13-15]. The app is designed to help all tobacco users, regardless of tobacco type(s) used. The app includes lessons and content on how to plan to quit and how to deal with cravings; it also includes tools for setting a quit date, developing a quit plan, and tracking when a participant lets urges pass. Furthermore, the app includes algorithm-based messages and notifications, such as helpful tips and reminders, and provides users the ability to text with trained coaches within the app. Information is provided about cessation medications and how to use them as part of the quitting process. Participants who successfully complete the core content and meet certain program milestones are issued a certificate of completion; however, all participants have continued access to all resources for up to one year.

### Sign-up Process

**Figure 2:**
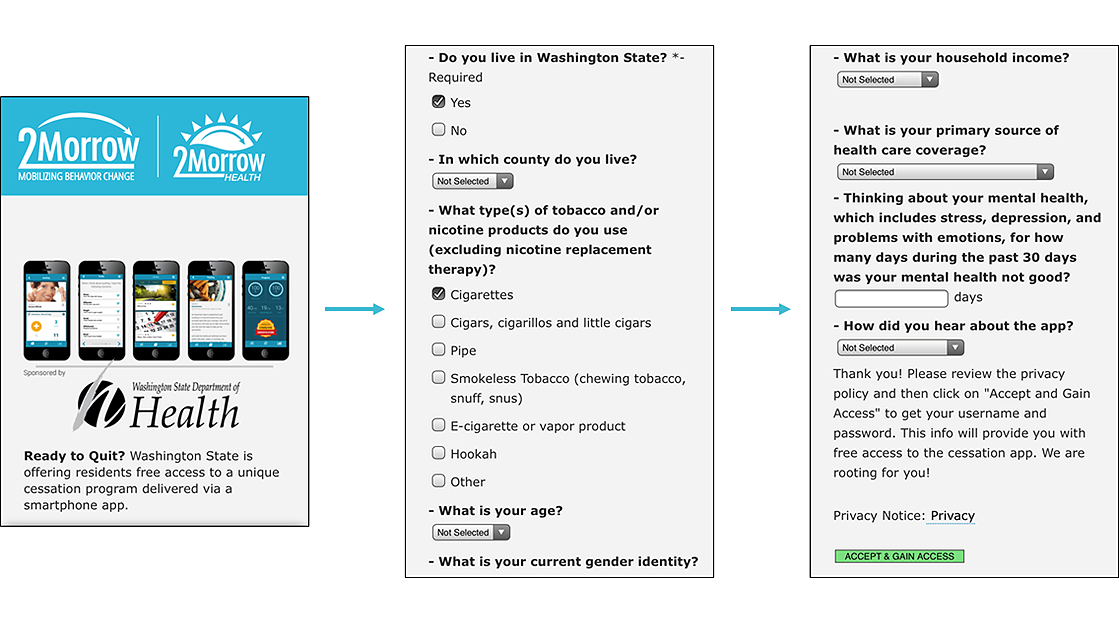
Excerpts from app sign-up page.

Promotional efforts varied throughout the 27 months for which sign-up data were examined, but generally relied upon the DOH website and business card–sized promotional materials distributed to the public through DOH tobacco prevention contractors. All promotional messaging directed prospective users to the DOH website using a short URL. This webpage linked them to the sign-up survey hosted on the 2Morrow website (Figure 2). For web browsers with the default language set to Spanish, the sign-up questions were automatically translated into Spanish. After completing the sign-up survey, participants were directed to review and accept the privacy policy to obtain a username and password, which would provide them with free access to the cessation app. Participants were informed of how their data might be used; the 2Morrow privacy policy read “We use de-identified information...to improve our programs and measure their impact and effectiveness to Users, and to conduct surveys and continue our research to improve our Services, which may result in published reports or articles.” Once participants signed up, 2Morrow provided them with a unique username and password combination that granted them free access to the app for 12 months upon download and activation.

### Data Collection and Measures

Demographic data were collected during sign-up. In addition to a question that requires prospective users to confirm that they live in WA, the sign-up survey includes as many as 16 optional demographic questions. Demographic data collected includes the following: age group, sex and gender, sexual orientation, race and ethnicity, education level, county, household income level, source of health care coverage (if any), mental health status, and type(s) of tobacco used. Prospective users who indicated a female sex assignment at birth were also asked if they are currently pregnant, planning pregnancy within the next 3 months, and/or breastfeeding.

Many questions were based on those used in the state’s Behavioral Risk Factor Surveillance System (BRFSS) surveys [6], so that the DOH could assess user representativeness of the broader WA adult tobacco user population. The BRFSS is an annual population-based, random-digit dial telephone survey of noninstitutionalized adults aged 18 years or older, and results are weighted to a variety of population characteristics. These questions, and others developed with input from state tobacco prevention community partners, were asked so that the DOH could also estimate its reach within certain populations. In 2020, the BRFSS combined landline and cell phone response rate was 47.4%. In the BRFSS, being a tobacco user is defined as being a current cigarette smoker, e-cigarette user, or smokeless tobacco user. Current cigarette smokers are respondents who report having smoked 100 or more cigarettes (or five packs) in their life and who report currently smoking every day or some days. Respondents who report currently using e-cigarettes or smokeless tobacco every day or some days are categorized as current e-cigarette and smokeless tobacco users, respectively. Use of multiple tobacco products is defined as currently using two or more tobacco products. More products were assessed during app intake than were assessed on the BRFSS, so there are two versions of multiple use among app users presented in Table 1; one version is the rate of using two or more of any of the available tobacco products assessed on the app, and the other only considers cigarettes, e-cigarettes, and smokeless tobacco for the sake of comparability to the BRFSS multiple tobacco product use rate.

Counties with a population of less than 100 persons per square mile and counties smaller than 225 square miles were categorized as rural [16].

**Table 1:** Characteristics of October 2018 to December 2020 tobacco cessation app users and 2020 Washington state (WA) tobacco users.

Characteristic		App users, N=1280		WA tobacco users			*P* value
		Value, n (%)	95% CI	Value (%)^a^	95% CI		
**Gender**							
	Female	780 (63.1)	60.3-65.8	42.4	39.5-45.4	<.001	
	Male	441 (35.7)	33-38.4	57.4	54.4-60.3	<.001	
	Nonbinary/other	15 (1.2)	0.7-2	N/A^b, c^	N/A	N/A	
	Not reported^d^	44	N/A	N/A	N/A	N/A	
**Age in years**							
	18-24	77 (6.5)	5.2-8	11.1	9.2-13.2	<.001	
	25-34	256 (21.6)	19.3-24	22.8	20.2-25.6	.50	
	35-44	349 (29.4)	26.8-32.1	19.7	17.4-22.2	<.001	
	45-54	298 (25.1)	22.7-27.7	16.8	14.8-18.9	<.001	
	55-64	173 (14.6)	12.6-16.7	16.9	14.9-19	.12	
	65 and up	33 (2.8)	1.9-3.9	12.7	10.9-14.6	<.001	
	Not reported	94	N/A	N/A	N/A	N/A	
**Educational attainment**							
	Less than high school	77 (6.3)	5-7.8	12.4	10.3-14.8	<.001	
	High school graduate or GED^e^	309 (25.3)	22.9-27.8	32.2	29.4-35	<.001	
	Some college	485 (39.7)	36.9-42.5	40.7	37.8-43.7	.62	
	College graduate	351 (28.7)	26.2-31.4	14.8	13-16.6	<.001	
	Not reported	58	N/A	N/A	N/A	N/A	
**Household income in US $**							
	Less than 15,000	270 (25.6)	23-28.3	10.2	8.6-12.1	<.001	
	15,000-24,999	189 (17.9)	15.6-20.4	17.6	15.3-20.1	.86	
	25,000-34,999	154 (14.6)	12.5-16.9	11.6	9.6-13.9	.05	
	35,000-49,999	129 (12.2)	10.3-14.4	14.8	12.7-17.1	.09	
	50,000-74,999	139 (13.2)	11.2-15.4	15.5	13.2-18	.14	
	75,000 or more	174 (16.5)	14.3-18.9	30.3	27.4-33.3	<.001	
	Not reported	225	N/A	N/A			
**Ethnicity and race**							
	Hispanic (all races)	78 (6.4)	5.1-7.9	7.2	5.7-9	.43	
	American Indian/Alaska Native (NH^f^)	21 (1.7)	1.1-2.6	3.3	2.3-4.5	.02	
	Asian (NH)	35 (2.9)	2-4	5.4	3.7-7.4	.02	
	Black (NH)	35 (2.9)	2-4	5.3	3.9-7	.01	
	Multiracial (NH)	60 (4.9)	3.8-6.3	3.2	2.4-4.2	.02	
	Native Hawaiian/Pacific Islander (NH)	11 (0.9)	0.5-1.6	1.3	0.7-2.1	.37	
	White (NH)	978 (80.3)	77.9-82.5	73.7	70.8-76.4	<.001	
	Not reported	62	N/A	N/A	N/A	N/A	
**Sexual orientation**							
	Heterosexual	1069 (87.5)	85.5-89.3	90.8	89-92.5	.01	
	LGBQA^g^	153 (12.5)	10.7-14.5	9.2	7.5-11	.01	
	Not reported	58	N/A	N/A	N/A	N/A	
**Mental health (MH)**							
	Less than 14 days of poor MH in the past month	715 (64.4)	61.5-67.2	74.4	71.6-77	<.001	
	14 or more days of poor MH in the past month	395 (35.6)	32.8-38.5	25.6	23-28.4	<.001	
	Not reported	170	N/A	N/A	N/A	N/A	
**Pregnancy status^h^**							
	Currently pregnant, planning pregnancy, or breastfeeding	42 (6.7)	4.9-9	N/A	N/A	N/A	
	Not currently pregnant, planning pregnancy, or breastfeeding	582 (93.3)	91-95.1	N/A	N/A	N/A	
	Not reported	169	N/A	N/A	N/A	N/A	
**Health insurance**							
	Employer or union health plan	412 (34.2)	31.5-36.9	41.6	38.6-44.7	<.001	
	Individual or family health plan	86 (7.1)	5.7-8.7	6.2	4.8-7.9	.41	
	Medicaid	356 (29.5)	27-32.2	18.8	16.5-21.3	<.001	
	Medicare	140 (11.6)	9.9-13.6	15	13-17.1	.01	
	None (uninsured)	147 (12.2)	10.4-14.2	12.5	10.5-14.8	.82	
	Other	31 (2.6)	1.8-3.6	2.6	1.8-3.5	>.99	
	TRICARE, Veteran’s Affairs, or military	34 (2.8)	2-3.9	3.3	2.2-4.7	.58	
	Not reported	74	N/A	N/A	N/A	N/A	
**WA geography**							
	Nonrural counties	861 (70.6)	67.9-73.1	75.1	72.7-77.3	.01	
	Rural counties	359 (29.4)	26.9-32.1	24.9	22.7-27.3	.01	
	Not reported	60	N/A	N/A	N/A	N/A	
**Products used^i^**							
	Cigarettes	1133 (90.9)	89.1-92.4	70.3	67.5-73	<.001	
	E-cigarette^j^ or vapor product	172 (13.8)	11.9-15.8	31.3	28.5-34.2	<.001	
	Smokeless tobacco	64 (5.1)	4-6.5	16.0	13.9-18.2	<.001	
	Multiple (2 or more of cigarettes, e-cigarettes, and smokeless tobacco)	155 (12.4)	10.6-14.4	15.6	13.4-18	.03	
	Multiple (2 or more of any product)	202 (16.2)	14.2-18.4	N/A	N/A	N/A	
	Cigars, cigarillos, and little cigars	74 (5.9)	4.7-7.4	N/A	N/A	N/A	
	Pipe	20 (1.6)	1-2.5	N/A	N/A	N/A	
	Other	32 (2.6)	1.8-3.6	N/A	N/A	N/A	
	None selected/not reported	33	N/A	N/A	N/A	N/A	

^a^Data in this column are from Washington state’s Behavioral Risk Factor Surveillance System (BRFSS) surveys. Absolute values are not provided.

^b^N/A: Not available.

^c^A nonbinary response option for this category was not provided by the BRFSS surveys.

^d^All percentages are calculated based on the number of participants who reported data for each category.

^e^GED: General Educational Development.

^f^NH: non-Hispanic.

^g^LGBQA: lesbian, gay, bisexual, pansexual, queer, questioning, or asexual.

^h^This question was only asked of the 793 participants who reported a female sex assignment at birth (not displayed).

^i^Participants were asked which type(s) of tobacco and/or nicotine products they use, and they could select multiple options; hookah tobacco is not displayed due to low response frequency.

^j^e-cigarette: electronic cigarette.

### Analysis

App data examined included individuals who signed up for the app between October 1, 2018, and December 31, 2020, and met program eligibility (verified living in WA). The analysis excluded individuals who did not activate the app by January 28, 2021, (n=1177), and 11 additional individuals who reported being less than 18 years old.

R software, version 4.0.4 (R Foundation for Statistical Computing) was used for data analysis, the R tidyverse package was used to generate frequencies and proportions for all variables, and exact confidence intervals for binomial proportions were estimated using the epitools package.

The WA 2020 BRFSS was used to generate prevalence estimates representative of the WA adult population of tobacco users. The total BRFSS sample size was 12,902. The R survey package was used to calculate weighted prevalence estimates and asymmetric 95% confidence intervals of demographic and risk factor distributions among current tobacco users. To compare BRFSS and app proportions, z-scores for two-sample means and two-sided *P* values were calculated and considered significant when *P*<.01, an approach consistent with the US Centers for Disease Control and Prevention (CDC) recommendations for assessing health disparities [17]. BRFSS estimates are not reported if the relative standard error exceeds 30%.

For both app user and BRFSS percentages, missing data were excluded from the analysis. Frequencies of missing values for optional demographic questions asked of app users at sign-up are presented as “Not reported” in Table 1.

## Results

The analysis included 1280 participants. Participants were primarily non-Hispanic White, identified as female, were between ages 25-54 years, had at least some college education, and reported a household income under US $50,000 (Table 1). Fewer respondents were from rural counties, identified as LGBQA, were uninsured, or were currently pregnant, planning pregnancy, or breastfeeding. Of the different types of tobacco and nicotine products participants were asked if they currently use, cigarettes were by far the most commonly reported, followed by e-cigarettes, cigars, and smokeless tobacco. Missing data ranged from a low of 2.6% (33/1280; types of tobacco and nicotine products used) to a high of 21.3% (169/793; pregnancy status among women).

Compared to BRFSS estimates of WA tobacco users, app users were significantly more likely to be female, age 35-54 years, non-Hispanic White or multiracial, LGBQA; they were also more likely to report poor mental health and live in a rural county (Table 1). College graduates, individuals reporting a household income of less than US $15,000 per year, and individuals with Medicaid insurance were also overrepresented (Table 1). Finally, tobacco users who report using e-cigarettes and/or smokeless tobacco products were far less likely to use the app than BRFSS estimates might suggest (Table 1).

## Discussion

### Principal Results

Overall, this study shows that a mobile tobacco cessation app reached a diverse population, including relatively large proportions of some groups disproportionately impacted by tobacco use; there was high representation of LGBQA individuals as well as individuals with low household income, on Medicaid insurance, with poor mental health, and residing in rural counties.

Research shows that 68% of smokers are interested in quitting [18] and that over 55% attempt to quit each year [18,19]. However, despite the existence of treatment options, less than one-third of smokers use these when trying to quit, and most use pharmacological support rather than behavioral support [18]. Furthermore, younger adults and certain ethnic and racial groups are less likely to seek help or use treatment [20]. Although there is a growing body of evidence evaluating [21,22] and testing the effectiveness of cessation apps [13-15,23,24], there is little known about who these apps can reach and benefit when offered, for free, to an entire population. Since most publicly funded tobacco cessation programs are designed to provide access to underserved communities and groups disproportionately impacted by tobacco use, both effectiveness and reach are important considerations for these public health interventions. This paper adds to this literature.

Lack of access to smartphone technology can serve as an obstacle to digital or app-based treatments. This is of concern for low-income populations who are less likely to own smartphones [25] and programs attempting to reach these underserved tobacco users. However, many of those who activated the app in our study also reported low incomes. These findings suggest that a mobile app for smoking cessation may be of relatively high interest among low-income populations.

Digital health cessation studies have shown that younger tobacco users are more likely to use web-based programs [26] as well as other digital programs, such as texting and mobile apps, than their older counterparts [27], who are more likely to call quitlines [28,29]. Our study demonstrates how states can complement their population-level cessation strategy with an app to reach and engage tobacco users in the quitting process relatively early in life. This is important because people who can quit smoking by age 40 gain 3 additional years of life expectancy compared to those who quit after age 50 [4].

This study benefited from large sample sizes, which allowed for the detection of frequent significant differences between the app user group and the BRFSS tobacco user population. In addition to individuals with low household income and 35-54-year-olds, the BRFSS comparisons revealed that LGBQA individuals, college graduates, those with poor mental health, those on Medicaid insurance, and those who reside in rural counties all activated the app in greater proportions than otherwise expected among the state’s population of tobacco users. As in comparable digital health cessation interventions and state quitline studies, there were also disproportionately high rates of use among tobacco users who identify as female and/or non-Hispanic White [27-29].

Of note, 1 in 15 women who used the app were pregnant, breastfeeding, or planning pregnancy, which inspired the DOH and 2Morrow to codevelop a tailored module of the app for this high-risk population [30], for whom other digital cessation interventions have demonstrated promise [31].

Offering a free cessation app may help state funders and other program sponsors reach these and potentially other priority populations who are known to have disproportionately high rates of tobacco use and tobacco-related disease. By offering cessation programs through different modalities, a telephonic quitline and a mobile app, the DOH can achieve broader demographic reach.

### Limitations

The generalizability of these results may be limited by a few factors. First, participants were not required to complete the demographic survey items to download and use the mobile app, which resulted in some missing data that could affect the interpretation of findings. Second, it is possible that participants could have registered and activated the app more than once across the study period, so some counts may be inflated. Third, the app was only promoted to WA residents, so the results may not generalize to other states or national populations. The DOH did not have any significant paid promotions for the app, which limits the conclusions about who might use the app, when robustly and continuously promoted. Additionally, 12 months into the app user data collection period for this study, the DOH and 2Morrow launched a vaping cessation program [32], the sign-up page for which was linked to the same webpage as the tobacco cessation app. Developed for teenagers and young adults who use e-cigarettes, this simultaneous offering may have reduced the number of app users who reported using e-cigarettes or vapor products in this study. Finally, both BRFSS and the app collected self-reported information which may be subject to systematic misclassification due to various reporting biases, such as social desirability or recall bias.

### Conclusions

The results from this study indicate that a diverse population of tobacco users, varying in terms of race, ethnicity, mental health status, sexual orientation, and other demographic characteristics, will use a free cessation app. Individuals who used the app in this study largely represent the demographic groups most at risk for cigarette smoking and associated premature disease and death. This may have implications for health equity. Understanding who uses cessation apps is important for developers and funders alike; this information can be used to address gaps in use, such as by developing marketing and outreach strategies or examining new product features that may be needed to appeal to different users. The extent to which users engage with the app should be explored and continuously improved upon to maximize effectiveness and, therefore, public health impact.

## Abbreviations

BRFSS: Behavioral Risk Factor Surveillance System
CDC: US Centers for Disease Control and Prevention
DOH: Washington State Department of Health
e-cigarettes: electronic cigarettes
GED: General Educational Development
HHS: US Department of Health and Human Services
LGBQA: lesbian, gay, bisexual, pansexual, queer, questioning, or asexual
NH: non-Hispanic
WA: Washington state
